# Rapid monitoring of reconstituted Wilms' tumor suppressor gene-specific t lymphocytes after allogeneic stem cell transplantation using quantitative real time Polymerase chain reaction

**DOI:** 10.1186/2051-1426-2-S1-P3

**Published:** 2014-02-24

**Authors:** Ahmad A Ab-Khader, Stefan Krause, Ernst Holler

**Affiliations:** 1 Department of Hematology and Oncology, University Hospital of Regensburg, Regensburg, Germany

## Background

Wilms' tumor suppressor gene (WT1) protein is over-expressed in some leukemias and various types of solid tumors, and it is considered to be an attractive target antigen for immunotherapy.

## Methods

After ex vivo stimulation of peripheral blood mononuclear cells (PBMCs) from 12 patients with two HLA-A2 restricted WT1 peptides (Db126 and WH187), between 220 days and 32 months after allogeneic SCT, interferon-gamma (IFN-γ) quantitative real time polymerase chain reaction (qRT-PCR) and ELISPOT assays were used to evaluate WT1 responses in 22 samples.

## Results

Both WT1 antigens induced significant quantities of IFN-γ mRNA production after 3 h in the qRT-PCR protocol. Among 18 samples of HLA-A2+, specific responses were detected in 27.8% (5/18) when Db126 peptide, compared to 16.7% (3/18) in the case of WH187 peptide. On the other hand, no detection (0/18) was obtained with ELISPOT, when both WT1 peptides were tested. In four HLA-A2- samples neither qRT-PCR nor ELISPOT detected any reconstituted WT1-reactive T cells in response to both peptides. As a result, the measurement of IFN-γ mRNA by qRT-PCR can be used to detect CTL responses 3 h after WT1 peptides stimulation of PBMCs.

## Discussion

In conclusion, qRT-PCR allows rapid monitoring of WT1-reactive CTLs reconstitution after allogeneic SCT using patient's PBMCs.

